# Assessing a fossil fuels externality with a new neural networks and image optimisation algorithm: the case of atmospheric pollutants as confounders to COVID-19 lethality

**DOI:** 10.1017/S095026882100248X

**Published:** 2021-11-16

**Authors:** Cosimo Magazzino, Marco Mele, Nicolas Schneider

**Affiliations:** 1 Department of Political Sciences, Roma Tre University, Roma, Italy; 2 The London School of Economics and Political Science, London, UK

**Keywords:** Air pollution, China, COVID-19, health, image learning, neural networks

## Abstract

This paper demonstrates how the combustion of fossil fuels for transport purpose might cause health implications. Based on an original case study [i.e. the Hubei province in China, the epicentre of the coronavirus disease-2019 (COVID-19) pandemic], we collected data on atmospheric pollutants (PM_2.5_, PM_10_ and CO_2_) and economic growth (GDP), along with daily series on COVID-19 indicators (cases, resuscitations and deaths). Then, we adopted an innovative Machine Learning approach, applying a new image Neural Networks model to investigate the causal relationships among economic, atmospheric and COVID-19 indicators. Empirical findings emphasise that any change in economic activity is found to substantially affect the dynamic levels of PM_2.5_, PM_10_ and CO_2_ which, in turn, generates significant variations in the spread of the COVID-19 epidemic and its associated lethality. As a robustness check, the conduction of an optimisation algorithm further corroborates previous results.

## Introduction

While not reflected in market prices, externalities of energy production induce adverse effects on the society that we cannot neglect anymore [1]. In most countries, transport, power generation, heating plants and industrial processes are heavily dependent on fossil fuels. Their combustion releases harmful pollutants into the atmosphere and drives the concentration of toxic molecules (notably PM_10_, PM_2.5_, O_3_, CO, NO_x_, SO_x_) in highly populated cities. Along with seminal studies, a strand of the literature emphasises that combusting fossil fuels produce externalities, which are yet known to affect the overall society through various economic, social and health effects. Long ignored, their associated costs are rarely accounted by environmental regulators and must be further internalised within energy planning.

Above all, agricultural outputs (notably wheat and rice) were found to be negatively influenced by short-lived climate pollutants and long-lived Greenhouse Gases (GHG), causing major crop yields and productivity losses [2–4]. In addition, air pollution was found to be associated with a decline in educational achievements and cognitive performance, disrupting the long-run human capital formation [5–8]. Moreover, air pollution was revealed as a substantial driver of overall crime and on several major crime categories, including those with economic motives [9–11], but also mental health issues [12–14].

Finally, a strand of literature has emerged, shedding light on the direct causal association between air pollutants concentration and health status. For instance, several studies have demonstrated that people exposed to elevated levels of fossil fuel-based air pollution are more likely to develop respiratory and cardiovascular diseases and present a lower life expectancy [15, 16]. Moreover, particle exposure has been pointed out to induce heart or lung disease, non-fatal heart attacks, irregular heartbeat, aggravated asthma, decreased lung function and increased respiratory symptoms such as irritation of the airways, coughing, or difficulty breathing [17, 18]. Indeed, PM_2.5_ can be inhaled and reach the deepest part of the lung and the circulatory system [19–21]. They travel into the blood and attain the cells, causing important damages and favouring long-run diseases [22]. Accordingly, toxic particle concentrations are at the heart of premature deaths in polluted cities and call for core solutions. For instance, Song *et al*. [23] estimated that when the concentration of PM_2.5_, PM_10_, NO_2_, O_3_ and CO increases by 10 μg/m^3^ in North China cities, the number of people hospitalised for hypertension raises by 0.56%, 0.31%, 1.18%, 0.40% and 0.03%, respectively. Goodkind *et al*. [24] analysed the Bitcoin economic, health and climate damages linked with air pollution emissions. The authors collected country-level data on emissions rates per kWh of electricity generation for four pollutants and combined them with knowledge on emission rates with the kWh of electricity usage per coin created. Knowing the average emissions released to generate a coin, they found that in 2018, each $1 of Bitcoin value created was responsible for $0.49 in health and climate damages in the US and $0.37 in China. Put differently, the human health and climate damages caused by Bitcoin represented *almost half* of the financial value of each US dollar of Bitcoin created (as represented by market prices). Xia *et al*. [25] employed a supply-driven input-output (I-O) model to estimate the monetary value of total output losses induced by air pollution-related disease across 30 Chinese provinces in 2007. They concluded that in 2007, the total reduced working time attributed to air pollution-related hospital admissions and deaths represent an economic loss estimated at around 346.26 billion Yuan (approximately 1.1% of the national GDP), which is almost the annual GDP of Vietnam in 2010. Tightly linked to this view, recent studies pointed out the role of air pollution in driving public and private healthcare expenditures [26–28]. Facing the above studies, critical questions can be raised about the well-established human activity-health-environment relationship in modern society.

Coronavirus disease-2019 (COVID-19) pandemic recently disclosed another potential externality from polluting emissions, beyond the well-known respiratory diseases: the possible susceptibility of human immune systems to virus contagions. Before the beginning of the outbreak, only a few studies linking air pollution to the spread of various viral infections were present in the literature [29–31]. However, due to its unprecedented nature, little is known on the atmospheric drivers of COVID-19. Along with the ongoing pandemic, a range of health risk factors involved in the spread of the epidemic has been commonly identified by researchers (i.e. older age, hypertension and respiratory issues) [32, 33]. However, facing the heterogeneous diffusion rate experienced by urban areas worldwide, some asked whether atmospheric conditions might be an additional driver to COVID-19 spreading and lethality.

A new research direction has been opened by seminal experimental studies in search of establishing a link between environmental and health indicators, distinguishing across countries and pollutants: Bashir *et al*. [34] on California; Setti *et al*. [35] on Italy; Travaglio *et al*. [36] on England; Yongjian on China; Magazzino *et al*. [37] and Mele *et al*. [38] on France; Magazzino *et al*. [39] on New York state; Mele and Magazzino [40] on India. As a result, all of them gave at least support to the existence of a substantial correlation between air pollution exposure and COVID-19 spread. However, some of the standard econometric procedures showed serious limitations in depicting causal inferences [41]. Indeed, the constraint of data unavailability makes the number of variables included into linear specifications limited, which, in turn, might report misleading estimates in absence of confounding factors. For the Hubei province in China, the epicentre of the pandemic, no study has been carried out. This is surprising since the critical pollution levels and the dramatic COVID-19 outbreak observed there make this case interesting. In China as in other developing countries, most of the population live in places with unsafe air [7]. The damages caused by air pollution imposes substantial health and economic costs that are not reflected in the market prices of fossil fuels. Therefore, while the exact predisposing factors contributing to worsen clinical severity and death of affected patients remain unclear, researchers unanimously argue that mitigating this primary pollutant ought to become a priority policy target. Accordingly, there is a need to understand how the rate of COVID-19 spread is affected by extreme disruptions in atmospheric pollutants as it can provide crucial clues to explain the efficiency of lockdown policies and helpful tools to control the epidemic diffusion in densely populated and polluted areas. Given that the COVID-19 pandemic is currently ongoing and far from being over, the complexity of such a topic requires urgent scientific responses.

In this research, we extend the discussion on the relationship between economic activity, atmospheric externalities derived from fossil fuel combustion and respiratory health outcomes, by bringing a specific focus on the channel between air pollution and COVID-19-related deaths. A potential mechanism is that particulate matters (PM_2.5_) can be inhaled and reach the deepest part of the lung and the circulatory system [19–21]. They travel into the blood and attain the cells. They cause lung cell inflammation, thereby increasing the sensitivity and damages of symptoms in COVID-19 patients [22, 42]. Starting from the recent evidence drawn in Magazzino *et al*. [37], this paper seeks to contribute to the literature in three distinct ways.

- First, this study fills a gap in the literature, conducting the first empirical assessment of the relationship between air pollution and COVID-19 related deaths for the case of the epicentre of the pandemic: the Hubei province in China. While the consumption of oil, natural gas and coal covers 87% of the total needs in this country, only 27% of the power sector is decarbonised, explaining why Chinese cities continue to record critical pollution levels^1^. Thus, critical levels of air pollution have been recorded in Chinese cities [45, 46].
- Second, this paper extends the novel literature that employed Machine Learning (ML) tools to assess the complex dynamic nexus between air pollution and virus epidemic [47–50]. As stated in Chudnovsky [51], some criticisms towards econometric-based inferences remain and should be overcome by more advanced methodological tools. Nonetheless, our study contrasts from the previous one as it applies a new sophisticated Deep Learning (DL) process derived from Artificial Neural Networks (ANNs) experiments for the first time.
- Third, this research relies on an original dataset elaborated based on a newly available series of local daily data on atmospheric pollutants (PM_2.5_, PM_10_ and CO_2_^2^) and COVID-19 status (cases, resuscitations and deaths) in China. All variables cover the largest and most recently available period of time (i.e. from January 20**^th^** to July 31**^st^**, 2020).

Besides the Introduction, the rest of the paper proceeds as follows: Section ‘The Revealing context of the externality: COVID-19 pandemic in Chinese polluted areas’ presents the scenario of the COVID-19 pandemic in Chinese polluted areas. Section ‘Literature review’ outlines the literature review. In Section ‘Data and methodology’ the theoretical ML framework is provided. Section ‘Results and discussion’ displays, comments and discusses the empirical results. Finally, in Section ‘Conclusions and policy recommendations’, concluding remarks are given, along with policy recommendations.

## The revealing context of the externality: COVID-19 pandemic in Chinese polluted areas

COVID-19 is a viral respiratory disease that can be contracted through three key channels: saliva, nasal discharge, or airborne particles [52]. When contracted, the disease induces a myriad of respiratory difficulties accompanied by fever and dyspnoea. First discovered in Wuhan, the COVID-19 virus has quickly spread across countries. By early 2020, the scale of the virus became worldwide, forcing countries to implement drastic and historical lockdown measures [53]. As each country began preventive and containment decrees, many citizens travelling abroad could not reach their home country while economic and social activities (i.e. the transport, tourism and hotel sectors) faced important limitations for national health safety reasons. Since the Spanish flu pandemic of 1918 (which killed about 50 million people worldwide), the current situation remains historical [54].

Nonetheless, before the shutdown of its manufacturing activities, Chinese cities have been recording critical air pollution levels that became of major concern for researchers and environmental organisations [55–57]. Due to its heavy dependence on the combustion of fossil fuels (i.e. for industrial and power generation purposes), the Chinese economy has driven the release of toxic polluting substances into the atmosphere for decades.

A wide range of empirical studies claims evidence that metropolitan cities in China significantly contribute to higher mortality rates in China. According to Aunan *et al*. [58], it has been estimated that polluting particles might be responsible for the yearly premature death of about a million people in this country (9% of the non-communicable diseases reported), concentrated in the most vulnerable population (elderly and/or displaying cardiovascular and respiratory issues). Being highly dependent on fossils fuels, the population around transport and industrialised areas might be more likely to contract respiratory issues including cancer (especially in locations where coal mining takes place) [59]. Accordingly, people with underlying health conditions and weak immune systems are more likely to present a higher risk of contracting the virus than those without health conditions or more robust immune systems.

Hover, as a result of the shutdown of economic and transport activities used to control Covid-19, Mountford [60] measured a 15% emissions reduction in the Chinese industrial sector, which remains lower than the worldwide average estimated at −26% by Le Quéré *et al*. [61]. This might explain the gradual decrease in the rates of deaths reported across cities like Wuhan, Shangai, Beijing and other provinces [59].

Far from being a coincidence, this highlights the possibility that reaching lower premature deaths levels can be achieved through reduced economic activities. More so, this underlines that the combustion of fuel creates new health externalities (i.e. the potential susceptibility of human immune systems to a virus contagion), recently revealed by the COVID-19 pandemic.

## Literature review

Notwithstanding its novelty, the determinants and consequences of COVID-19 have been the subject of extensive assessments. Within this literature, a strand has emerged assessing the environmental determinants of COVID-19 spread. Atmospheric factors and the concentration of harmful pollutants, in particular, have concentrated intensive discussions. However, despite sharing a common research question, one will see that approaches contrast (i.e. mathematical modelling, virus diffusion simulation, econometric regressions, ML experiments) and the case studies differ (cities, regions, single-country, multi-locations). In the first part, we outline the papers which examined the air pollution-COVID-19-related deaths nexus for regions located in various countries, except China (*2.1*.). Then, we summarise the main assessments on the single case of Chinese cities (*2.2*).

### General assessments of the air pollution-COVID-19 nexus

Coccia [62] collected data on 55 Italian cities which are provincial capitals and suggested that the diffusion of COVID-19 in cities with high levels of air pollution generated higher numbers of COVID-19 related infected individuals and deaths. More specifically, results reveal that the number of infected people is significantly higher in cities where the limits of PM_10_ or ozone are exceeded over 100 days per year and the average wind speed and temperature level are lower. In Magazzino *et al*. [63], the authors used ANNs experiments in an ML framework on Brazilian series. They showed that more intensive use of renewable energy could generate a positive GDP acceleration, which in turn, could offset the harmful pollution-COVID-19 association. Using a slightly different approach, Magazzino *et al*. [39] inspected the case of New York state using city-level daily data and two ML experiments. PM_2.5_ and NO_2_ were found to be the most significant pollutant agents responsible for facilitating COVID-19 attributed death rates. Going one step further, Mele *et al*. [38] estimated that the threshold values of NO_2_ connected to COVID-19 range between 15.8 μg/m^3^ for Lyon, 21.08 μg/m^3^ for Marseille and 22.9 μg/m^3^ for Paris. Finally, these results are in line with those of Travaglio *et al*. [36] for England; Bashir *et al*. [34] for California and a set of Italian examinations: Zoran *et al*. [64] for the city of Milan; Setti *et al*. [35] for eight Italian regions; Conticini *et al*. [65] for northern Italy; Fattorini and Regoli [66] for 71 Italian provinces; and Frontera *et al*. [67] for the major Italian regions. On a more global scale, this corroborates the findings of Razzaq *et al*. [68] for the top 10 affected states of the U.S., but also Vasquez-Apestegui *et al*. [69] for 24 districts of Lima (Perù). Finally, Sarkodie and Owusu [70] demonstrated that high temperature and high relative humidity reduce COVID-19 cases, deaths and improve recovery using series covering 20 countries. Reciprocally, low temperature, wind speed, dew/frost point, precipitation and surface pressure prolong the activation and infectivity of the virus. At the city level, Sasidharan *et al*. [71] designed a preliminary assessment on the linkage between short-term NO_2_ concentration and COVID-19 cases and fatality rates for the case of London (UK). Based on data up to March 31^st^ 2020, the COVID-19 fatality rate was found positively correlated with short-term NO_2_ pics. Nonetheless, Saez *et al*. [41] contrast with these studies as they showed that, although some mechanisms may explain this air pollution-COVID-19 dynamic, the spatial spread in Catalonia (Spain) might be more attributed to population interactions rather than a chronic immune sensitivity to this virus. Finally, the latest evidence on this channel can be found in Konstantinoudis *et al*. [72] for England; Liu *et al*. [73] for California; Coccia [74] for Italian regions; Coccia [75] for a global sample of 160 countries.

With a slightly different approach, Haque and Rahman [76] relied on linear regression models and concluded that high temperature and humidity are coupled with a significant reduce COVID-19 transmission reduction in Bangladesh. Such evidence is in line with those of Mele and Magazzino [40] who drew similar conclusions for India. Following this view, Rosario *et al*. [77] highlighted that there is a substantial negative correlation between solar radiation and COVID-19 incidence in the State of Rio de Janeiro (Brazil), whereas negative inferences are indicated when looking at temperature and wind speed. A review on the association among meteorological factors, air pollution and COVID-19 can also be found in Magazzino *et al*. or Srivastava [78]. Table 1 summarises the main information of this novel literature.

**Table 1.** Previous air pollution-COVID-19 assessments, excluding the Chinese case

Author(s)	Country	Sample period	Air pollution indicator(s)	Relationship between air pollution on COVID-related deaths
Bashir *et al*. [34]	California (US)	4 March 2020–24 April 2020	CO, NO_2_, Pb, PM_2.5_, PM_10_, SO_2_, VOC	Yes
Coccia [62]	55 Italian provinces	March–May 2020	PM_10_	Yes
Conticini *et al*. [65]	Northern Italy	15 March 2020 onward	NO_2_, O_3_, PM_2.5_, PM_10_, SO_2_	Yes
Fattorini and Regoli [66]	71 Italian provinces	Data up to 27 April 2020	NO_2_, PM_2.5_, PM_10_	Yes
Frontera *et al*. [67]	Italian regions	Data up to 31 March 2020	PM_2.5_	Yes
Magazzino *et al*. [39]	New York state (U.S.)	3 March –26 June 2020	NO_2_, PM2_.5_	Yes
Magazzino *et al*. [37]	Paris, Marseille, Lyon	18 March 2020–27 April 2020	PM_2.5_, PM_10_	Yes
Mele *et al*. [38]	Paris, Marseille, Lyon	18 March 2020–27 April 2020	NO_2_	Yes
Mele and Magazzino [40]	25 major Indian cities	29 January –18 May 2020	CO_2_, NO_2_, PM_2.5_	Yes
Razzaq *et al*. [68]	10 US States	29 February 2020–10 July 2020	O_3_	Yes
Saez *et al*. [41]	Catalonia (Spain)	25 February 2020–16 May 2020	NO_2_, PM_10_	No
Sasidharan *et al*. [71]	London (UK)	data up to 31 March 2020	NO_2_, PM_2.5_	Yes
Setti *et al*. [35]	8 Italian regions	10 February 2020–29 February 2020	PM_10_	Yes
Travaglio *et al*. [36]	120 sites in England	1 February 2020–8 April 2020	NO_2_, NO_x_, O_3_	Yes
Vasquez-Apestegui *et al*. [69]	24 districts of Lima (Perù)	Data up to 12 June 2020	PM_2.5_	Yes
Zoran *et al*. [64]	Milan (Italy)	January 2020–April 2020	NO_2_, O_3_	Yes
Konstantinoudis *et al*. [72]	England	Up to 30 June 2020	NO_2_, PM_2.5_	Yes
Liu *et al*. [73]	California	26 January 2020–7 May 2020	NO_2_	Yes
Coccia [74]	Italian regions	Up to 23 June 2020	O_3,_ PM_2.5,_ PM_10_	Yes
Coccia [75]	160 countries	Up to 2021	PM_2.5_	Yes

*Source*: our elaborations.

*Notes*: ‘Yes’ means that the existence of a significant association between air pollution levels and COVID-19 cases/mortality was established. ‘No’ indicates that no significant relationship was supported among indicators.

### Studies On the air pollution-COVID-19 nexus in China

A few assessments have tackled the single case of China and associated findings suggested that metropolitan cities in China contribute significantly to higher mortality rates in China as many develop respiratory-related issues from exposure to polluted air. For instance, Shen *et al*. [79] argued that critical atmospheric pollution events were frequently observed during the strictest lockdown in Hubei. Zhang *et al*. [80] applied a mathematical model with multiple datasets to estimate the transmissibility of the COVID-19 virus and the severity of the illness associated with the infection and how both were affected by unprecedented control measures. The analyses show that before 19^th^ January 2020, 3.5% of infected people were detected; this percentage increased to 36.6% thereafter.

Xu *et al*. [81] collected data on three cities in the Hubei province and supported that during February 2020, when the epidemic prevention and control actions were taken, the average concentrations of atmospheric PM_2.5_, PM_10_, SO_2_, CO and NO_2_ in the three cities were 46.1 μg m^–3^, 50.8 μg m^–3^, 2.56 ppb, 0.60 ppm and 6.70 ppb and were 30.1%, 40.5%, 33.4%, 27.9% and 61.4% lower than the levels in February 2017–2019, respectively. Going one step further, Yongjian compiled data on 120 Chinese cities ranging from 23^rd^ January to 29^th^ February 2020. A Generalised Additive Model (GAM) is applied on a framework and associated findings claimed evidence of a positive and significant association between PM_2.5_, PM_10_, NO_2_ and O_3_ concentrations with COVID-19 confirmed cases in this country, which is in line with Yao *et al*. [82, 83] for the city of Wuhan (China). Finally, Gupta *et al*. [84] collected data on nine cities in Asia (including three in China) and provided evidence supporting the existence of a correlating relationship between PM_2.5_ and PM_10_ and COVID-19-related deaths. A neighbouring conclusion can be found in Xie and Zhu [85] who indicated that temperature has a positive linear relationship with the number of COVID-19 cases with a threshold of 3 °C in 122 cities from China. Nonetheless, no evidence supporting that case counts of COVID-19 could decline when the weather becomes warmer was provided. Table 2 outlines the main information of this Chinese-related literature.

**Table 2.** Previous air pollution-COVID-19 assessments in China

Author(s)	Country	Sample period	Air pollution indicator(s)	Relationship between air pollution on COVID-related deaths
Xu *et al*. [81]	Three Chinese cities	2017–2019	PM_2.5_, PM_10_, NO_2_, O_3_	Yes
Yao *et al*. [82, 83]	Wuhan (China)	19 January 2020–15 March 2020	PM_2.5_, PM_10_	Yes
Yongjian	120 Chinese cities	23 January 2020–29 February 2020	CO, NO_2_, O_3_, PM_2.5_, PM_10_, SO_2_,	Yes
Gupta *et al*. [84]	Nine cities from Asia (India, China, Pakistan and Indonesia)	Up to 2 July 2020	PM_2.5_, PM_10_	Yes

*Source*: our elaborations.

*Notes*: ‘Yes’ means that the existence of a significant association between air pollution levels and COVID-19 cases/mortality was established. ‘No’ indicates that no significant relationship was supported among indicators.

Lastly, Kucharski *et al*. [86] provided specific analysis of the city of Wuhan but excluded pollution series from their framework. Instead, they fitted a stochastic transmission dynamic model to four datasets and underlined that in locations with similar transmission potential to Wuhan in early January 2020, the likelihood that the infection spreads within the population reached 50% if at least four independent COVID-19 cases are introduced within the total sample. In Sarkodie and Owusu [87], the authors revealed that the effect of confirmed cases on the novel coronavirus attributable deaths is heterogeneous across the 31 Provinces/States in China. More specifically, an increase in confirmed cases by 1% increases coronavirus attributable deaths by ~0.10%–~1.71% (95% CI). All in all, further neighbouring COVID-19-related investigations can be found in Balsalobre-Lorente *et al*. [88] who examined the effects of economic and social isolation as dimensions of globalisation and brought fresh evidence on the effect of the isolation phenomenon due to the COVID-19 outbreak on the Chinese economic performance. In the same vein, Alola *et al*. [89] employed the empirical Markov switching regression approach to identify the first causal channels among the US financial stress situation resulting from the effects of COVID-19 daily deaths, COVID-19 daily recovery and the global economic policy uncertainty.

## Data and methodology

### Data Collection and empirical strategy

To assess the relationship among COVID-19 deaths, air pollution and economic growth in China (Hubei area), we collected daily data at a city level for the period from 20 January to 31 July 2020.

The study here focuses on a case study of the Chinese province of Hubei, the first area in the world to experience a rapid increase in confirmed cases of COVID-19 and related deaths. Sources of data are the Chinese Center for Disease Control and Prevention (http://www.chinacdc.cn/) for numbers of infected people and deaths; the Hubei Environmental Protection Agency (http://sthjt.hubei.gov.cn/) for levels of air pollution; and the institutional website of the three main cities of Hubei province for per capita economic growth series (en.yichang.gov.cn; en.xiangyang.gov.cn; en.whuan.gov.cn).

To generate a daily time-series relating to the variable of economic growth, we used the monthly and standardised data for the number of days. This procedure is suitable for an ML process. In fact, the machine interprets the data not as a time series, but as an aggregate of data. Each variable was transformed into logarithms (*ln*), first differences (*d*), squared (*s*) and logarithmic differences (*d.ln*). Thus, we have a dataset with 8100 observations. The large number of observations justify the choice of an experiment in DL.

ML studies have been an exponential growth in the last years, receiving interest from industry, academia and popular culture. These are driven by breakthroughs in ANNs, a set of techniques and algorithms that enable computers to discover complicated patterns in large datasets. Feeding the breakthroughs is the increased access to data (‘big data’), user-friendly software frameworks and an explosion of the available computing power, enabling the use of NN that is deeper than ever before [90].

Recently, due to the optimisation of algorithms, the improved computational hardware and access to a large amount of imaging data, DL has demonstrated indisputable superiority over the classic ML framework. DL is a class of ML algorithms that uses ANN architectures that bear resemblance to the structure of human cognitive functions. It is a type of representation learning in which the algorithm learns a composition of features that reflect a hierarchy of structures in the data [91].

Following Gardner and Dorling [92], Magazzino *et al*. [93–95] and Mele and Magazzino [96] we performed a DL experiment. NNs work in parallel and are, therefore, able to process a lot of data simultaneously and autonomously.

On the contrary, in standard or econometric statistical processes, each data is treated individually and/or in time series. Even though each neuron is relatively slow, parallelism partly explains the faster speed of the brain in performing tasks that require the simultaneous processing of a large number of data. In essence, it is a sophisticated statistical system with excellent noise immunity; if some units of the system were to malfunction, the network as a whole would have reduced performance but would hardly encounter a system crash. To do so, we used Oryx, a software that emerged as a power tool able to identify and analyse causal linkages in a multivariate setting. Adequate applications on neighbouring topics can be found in Didelez *et al*. [97], Sparks *et al*. [98] and Gärdenfors and Lombard [99].

The features of an ANNs model are the following:

- the development of the ‘neuron system’ is distributed over many elements. In other words, many neurons do the same thing;
- an address identifies each data of the algorithm used (a number), which is used to retrieve the knowledge necessary to perform a certain task;
- ANNs, unlike standard econometric models and their software, do not have to be programmed to perform a task. ANNs learn independently, based on experience or with the help of an external instructor.

**Fig. 1.**
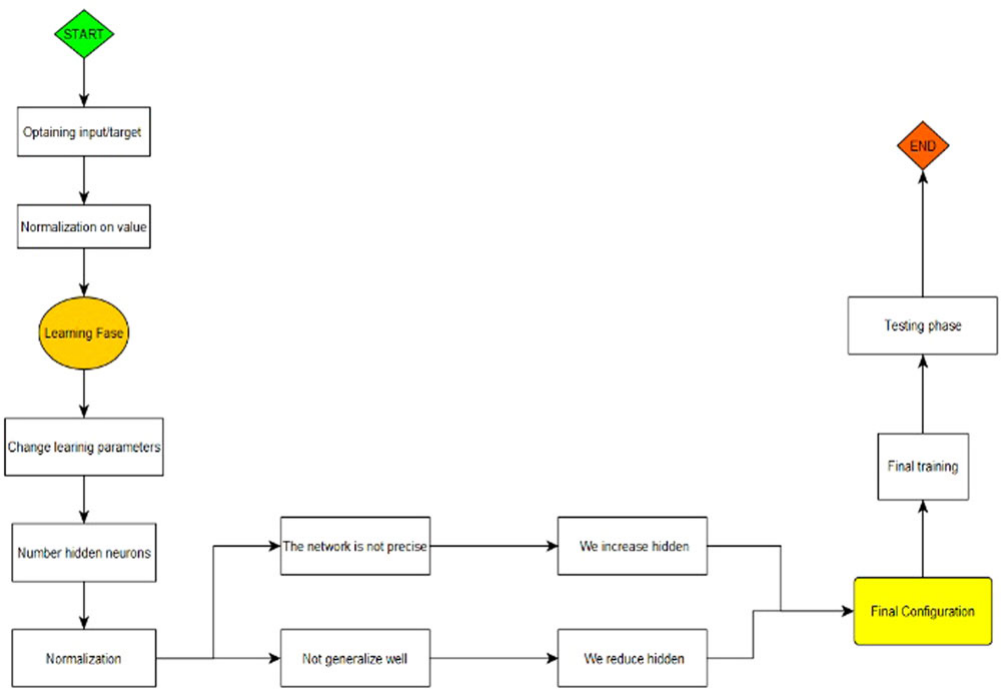
The ANNs process. *Source*: our elaborations in YeD.

The use of ANNs, as a subset of the ML tools, follows a precise implementation scheme for building the network and obtaining results. In Figure 1 we synthesised the process.

### Mathematical process on NNs

In what follows is described the theoretical process of the NNs experiments.1
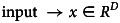
2
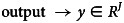
3
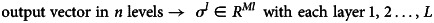
4
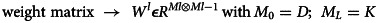
5
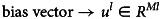
6
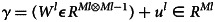
7
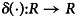


Our NN can be written as:8
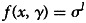
9
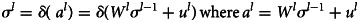
10
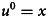


The activation functions can be linear or nonlinear, with *L* *=* *n*. In the first case:11
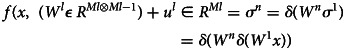


If *δ* is a linear function → *δ*(*u*^*l*^) = *k*_*l*_*u*^*l*^ where 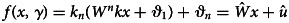


If we choose, in an arbitrary way, to use a non-linear activation function, we have:12
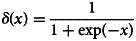
13
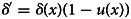
14
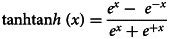
15
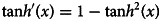


Thus, with rectified linear unit, we have:16
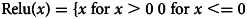


In our NN, MSE will be:17
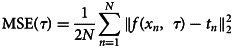


In (17):18
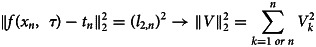


Now, the Log-Likelihood (LL) will be:19
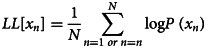


Therefore, after uploading the dataset in Oryx, we adapted the data series to an ML process through logarithmic and differential transformations. In fact, the higher the volume of data, the greater the capacity of analysis of the automatic intelligence system. The values are then normalised through the training selection procedure, which also begins a first test phase. In this phase, we evaluate the presence of omitted variables or outliers, which might cause false values of the NN. Subsequently, starting from the results obtained with the first test, we choose the suitable number of hidden neurons that are an integral part of the network. The next step is to launch the algorithm to create the NN. The result obtained is carefully analysed and we verify the precision and generalisation of the obtained network.

Then, we can proceed with the generation of a multivariate NN by targeting the number of deaths caused by COVID-19. We try to verify if the process of economic growth – connected to the pollution levels – represents a random acceleration of deaths from COVID-19. The following inputs are used: economic growth (*GDP_p*, *lnGDP_p*, *dGDP_p*, *sGDP_p* and *d.lnGDP_p*); pollution (*PM2.5*, *lnPM2.5*, *dPM2.5*, *sPM2.5*, *d.lnPM2.5*, *PM10*, *lnPM10*, *dPM10*, *sPM10*, *d.lnPM10*, *CO2*, *lnCO2*, *dCO2*, *sCO2*, *d.lnCO2*). The target are COVID-19 deaths (*Deaths*, *lnDeaths*, *dDeaths*, *sDeaths*, *d.lnDeaths*).

Once the NN has been built and the levels of inputs *vs.* targets calculated, we test the result through a model of relationships between variables via the Adaptive moment estimation (Adam) optimisation algorithm. In this way, two different estimation models are tested.

## Results and discussion

In the first experiment, we test the predictive capacity of 20 inputs concerning 8100 combinations on 5 targets. We adapt the NNs algorithm to predict the probability that each variable might cause a variation among the same variables.

**Fig. 2.**
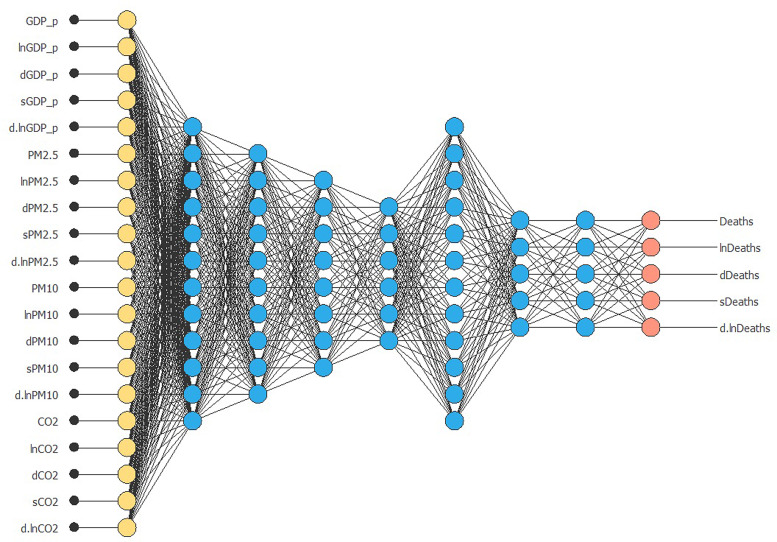
NNs model. *Source*: our elaborations in Oryx 2.0.8.

**Fig. 3.**
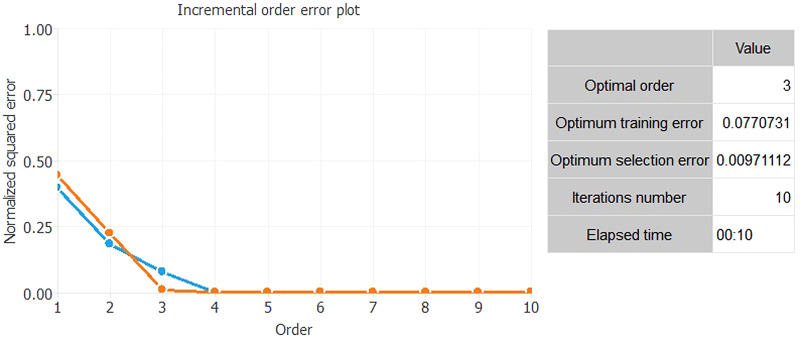
Incremental Order error test. *Source*: our elaborations in Oryx 2.0.8.

**Fig. 4.**
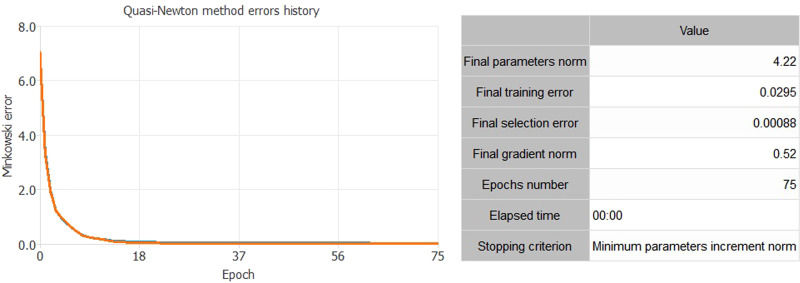
Quasi-Newton method algorithm. *Source*: our elaborations in Oryx 2.0.8.

Figure 2 shows the result of the NN analysis. It contains a scaling layer, a NN and an unscaling layer. The yellow circles represent the scaling neurons, the blue circles the perceptron neurons and the red circles the unscaling neurons. The number of inputs is 20, while the number of outputs is 5. The complexity, represented by the numbers of hidden neurons, is 12:10:8:6:12:5:5. The construction of the NN is validated through two complex tests (Figs 3 and 4). The Incremental Order error test hypothesises the presence of numerous better alternative NN models than the selected one. In particular, this test defines several errors (testing and training) generally lower than the NN of the selected model. Thus, if there is a better model than the estimated one, the test shows a high level of errors in both training and testing. The second model is based on the Minkowski error, concerning the Quasi-Newton method algorithm. This method computes an approximation of the inverse Hessian at each iteration of the algorithm, by only using the gradient information.

As we can see from Figures 3 and 4, both tests confirm the strength of the constructed NN. The test in Figure 3 shows the absence of an alternative NN to the constructed one. The second test in Figure 4 shows that the error in the transmission of the NN, with the growth of the gradients descendent, is gradually lower and tending towards zero.

Therefore, we can now generate the results of NN signal prediction. This system represents a pure DL image model. In other words, simulating an NN, the model shows how the signals are interconnected with each other. This result represents a causal link between thousands of combinations, which show the effect towards one of the five targets, without the operator's intervention.

**Fig. 5.**
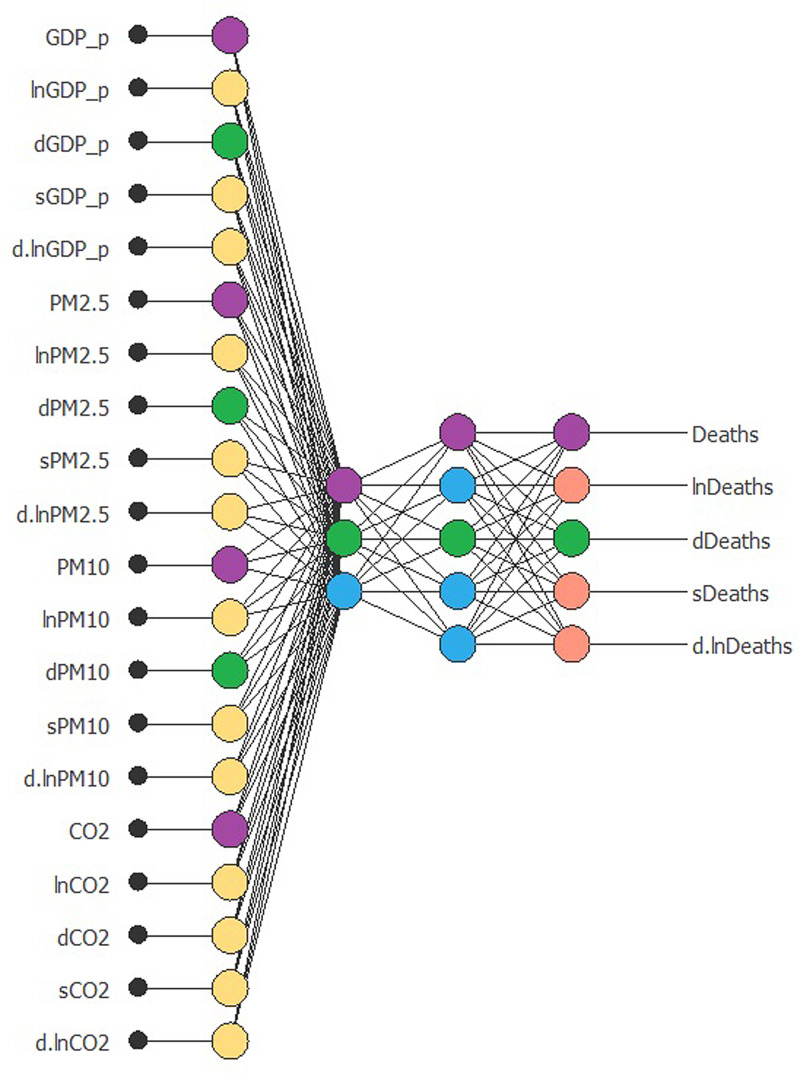
DL image results. *Source*: our elaborations in Oryx 2.0.8.

**Fig. 6.**
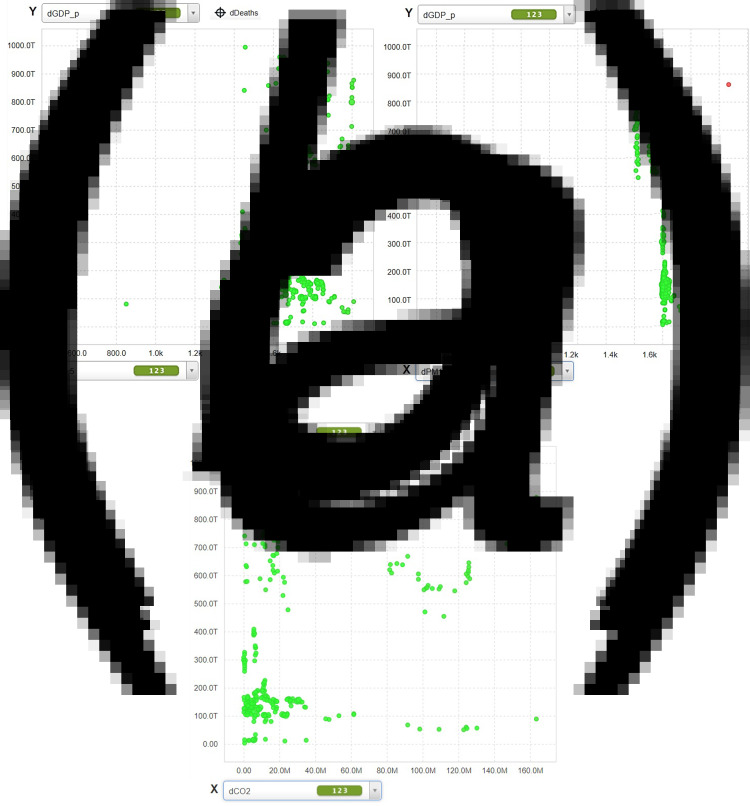
Image optimisation on GDP, PM_2.5_, PM_10_ and CO_2_ growth rates. (a) Relationship between GDP and PM_2.5_ (b) Relationship between GDP and PM_10_ (c) Relationship between GDP and CO_2._ *Notes*: dGDP_p: GDP growth rate; dPM2.5: PM2.5 growth rate; dPM10: PM10 growth rate; dCO2: dCO2 growth rate. *Source*: our elaborations in Oryx 2.0.8 and BML.

Figure 5 describes the results of the model. The NN has two colours that describe the predictive causal relationship from inputs to outputs. The first purple-coloured circle represents the transmission of the NN from stationary inputs to a stationary target. In fact, we can observe that per capita GDP, PM_2.5_, PM_10_ and CO_2_ meet in a top-level neuron layer. This situation suggests the presence of interconnections among these variables. The joint signal of the inputs meets, after a higher neural level, the target *Deaths*. In other words, the COVID-19 deaths in Hubei were affected by the level of pollutants in the environment that would have favoured the process of deaths in the area under study. However, this result is enriched by the analysis of the green circles. Usually, an NN model signalled by images does not highlight joint processes. Instead, no further analysis elements were generated in this case. The green circles represent a prediction model in the NN. Input variables are all first differences. We note how the variation in per capita GDP meets the variation of fine particulates in an early neural stage. In this situation, CO_2_ is absent. The fine particulates, inevitably generated by the process of economic growth and greater use of energy, generate the variation of deaths from COVID-19. This result is significant. We can state that the continuous emission of fine particles increases the probability of generating deaths from viral pandemics in the future. In order to test the results obtained through a different model, we use the image optimisation algorithm. In particular, we investigate whether future variations of COVID-19 deaths in Hubei province can be caused by a relationship between economic growth and pollution (Fig. 6).

As we can see from Figure 6, the results partially confirm those of the NN. In fact, we can see that, by targeting *dDeaths*, PM_2.5_ and PM_10_ are closely linked to the change in economic growth. As per capita income grows, we notice an increase in the variation of both PM_2.5_ and PM_10_ particulates and in turn, these variables generate the variance of *dDeaths*. On the contrary, this predictive situation does not exist in the figure concerning *dCO_2_*. In fact, as per capita income increases, CO_2_ growth is evident only in the initial phase of the optimisation process. This result underlines, however, how pollutants are generated by economic growth and as a whole (see Figure A and Figure B in the Appendix).

In sum, the DL outcomes slightly connect with the most recent literature on this topic. One will see that some disparities arise from the literature, as a minor share of studies displays conflicting conclusions, which may be caused by the heterogeneity of modelling methods employed so far. Our results extend the empirical findings presented in Magazzino *et al*. [37, 39] and Mele *et al*. [38], since both assessments, despite focusing on different case studies, revealed the existence of a clear relationship among economic activity, polluting particles and deaths due to the COVID-19 pandemic using ANNs experiments and a Causal Direction from Dependency Algorithm (D2C). In particular, our findings echo those drawn from an Ecological Regression Analysis in Wu *et al*. [100, 101]. The authors fitted a negative binomial mixed model using COVID-19 mortality rates as the outcome and long-term average PM_2.5_ as the exposure of interest, adjusting for 20 county-level covariates. They suggested that one microgram per cubic metre of PM_2.5_ is associated with a 15% increase in the mortality rate due to the COVID-19 in the US. Such inferences are also in line with Becchetti *et al*. [102, 103] who used econometric correlation methods to claim evidence of a direct link between air quality and COVID-19 lethality, especially for people displaying predisposition to pulmonary pathologies. Finally, our ML inferences extend the econometric evidence revealed in Razzaq *et al*. [68] for 10 US states; Bashir *et al*. [34] for California; Travaglio *et al*. [36] for 120 sites in England; and Zoran *et al*. [64] for Milan. Finally, our results corroborate the latest evidence on this channel supplied in Konstantinoudis *et al*. [72] who used spatial Bayesian hierarchical models for England; Liu *et al*. [73] who used a time-series analysis for California; Coccia [74] who used linear estimation regression for Italian regions; Coccia [75] who used Independence Sample Tests for 160 countries. However, our estimates conflict with Saez *et al*. [41] who set up a mixed longitudinal ecological design with a Generalised Linear Mixed (GLM) model for located areas in Catalonia. They concluded that some biological mechanisms may explain, at least partially, the association between long-term exposure to air pollution and COVID-19 lethality, they argued that the spatial diffusion of the virus should be linked to population density, mobility across locations and age than atmospheric factors. Congruent with our conclusions, Ogen reviewed data from the ESA Sentinel 5P satellite and mapped the distribution of nitrogen dioxide in Europe in the months leading up to the pandemic. The author collected the information supplied by a satellite TROPOspheric Monitoring Instrument (TROPOMI) and compared the mean deaths cases and the percentage of deaths in each NO_2_ concentration range. Results demonstrated that 78% of the deaths from COVID-19 were concentrated in highly Nitrogen polluted Italian and Spanish areas. Looking at the Chinese-related literature, the present findings do support those of who collected daily atmospheric measures (PM_2.5_, PM_10_, SO_2_, CO and O_3_) in 120 Chinese cities and concluded the existence of a causal link between air pollution and COVID-19-related deaths. Going one step further, Yao *et al*. [82, 83] exited from the particulate matter-related inferences and brought a specific focus on NO_2_. In final, they drew the same conclusion for 63 Chinese cities, which again, corroborates the previous literature as well as our results and echo to the latest evidence supplied in Gutpa *et al*. [84] for 9 Asian cities.

The hypothesis that the dust suspended in the atmosphere can convey the virus inside the respiratory tract is a plausible situation but requires in-depth studies. We are convinced that it is a possible phenomenon, but unlikely due to airborne viruses' concentration levels in outdoor environments. On one hand, the nature of the indoor environment (i.e. private housing) limits the number of contaminated droplets emitted by an infected person – from 100 to 10 000 per litre of exhaled air, as they are rapidly dispersed into the ambient air. On the other hand, it is known that prolonged exposure to high concentrations of pollutants, particularly fine dust, causes respiratory and cardiovascular diseases, which can likely aggravate the clinical picture of the infected. It is yet known that COVID-19 determines a rapid and significant increase in the inflammatory response, which can involve the blood vessels and the heart. This situation increases the likelihood of critical events, such as vasculitis and myocarditis, which in turn, increases the likelihood of heart attack and death [104]. It has been pointed out that the mortality from COVID-19 is larger for people presenting vulnerable co-pathologies: cardiovascular disease, hypertension, chronic breathing disorders and cancer. We can, therefore, speak about a probable relationship between pollution and marked mortality of COVID-19 infection.

## Conclusions and policy recommendations

In this paper, we show that the combustion of fossil fuels for transport and power purposes has health implications that go beyond the well-established respiratory and cardiovascular issues. While it is known that the generation of carbon-intensive energies releases harmful particles in the atmosphere, much less is known on the other side of the channel. Here, our underlying hypothesis is that elevated concentrations may potentially render the human immune system more susceptible to a virus contagion and thus favour the spread of the epidemic [102, 103][37]. As a matter of fact, clear channels have been highlighted in the medical literature for a wide range of infections [105], but the case of the severe acute respiratory syndrome coronavirus 2 (SARS-Cov-2) remains overlooked. Particle exposure has been pointed out to induce heart or lung disease, non-fatal heart attacks, irregular heartbeat, aggravated asthma, decreased lung function and increased respiratory symptoms such as irritation of the airways, coughing, or difficulty breathing [17, 18]). PM_2.5_ can be inhaled and reach the deepest part of the lung and the circulatory system [19–21]. A potential mechanism is that particulate matter passes into the blood and attain the cells. They cause lung cell inflammation, thereby increasing the sensitivity and damages of symptoms in COVID-19 patients [22, 42].

Based on this background, our paper tests the above hypothesis for PM_2.5_ and PM_10_ and extend the experiment to CO_2_ elements. It tests whether fine particulates and carbon dioxide derived from fuel combustion can be identified as effective contributors to COVID-19 spread and lethality. To do so, we take the Chinese Hubei Province, known as the epicentre of the pandemic, as an illustrative case to assess the above complex nexus between environmental and health indicators (*i*) and draw insights to complement the global state of knowledge on this topic (*ii*). Based on this original case study, a sophisticated ML approach is adopted, applying an image NN model on newly available daily time series. This includes economic activity, a range of atmospheric pollutants and COVID-19 indicators (cases, resuscitations and deaths). Related empirical results are highly insightful. The NNs model identified two significant pathways for the neural signal. Above all, a first is found to operate from economic growth, PM_2.5_ and PM_10_ to the level of COVID-19-related deaths. Furthermore, a second linkage is depicted and highlights how the variation in COVID-19-related deaths is significantly influenced by changes in the growth rate of the economic activity on one hand and pollutants' concentrations, on the other hand. As a robustness check, the conduction of an optimisation model algorithm corroborated these causal inferences. It emphasises how changes in economic growth affect the changes in fine particulates, which, in turn, generate variations in the number of deaths associated with COVID-19. But beyond corroborating the polluting particles-health outcome literature, our results suggest that the COVID-19 crisis revealed an additional pollution-related cost, underestimated so far in urban areas. In this study, we corroborate the rising literature suggesting that the combustion of fossil fuels affect health through an additional channel: the spread and lethality of COVID-19. Thus, our findings contribute to shed light on the existence of non-negligible empirical connections among economic activity, atmospheric pollution and COVID-19 lethality in the Hubei Province, China.

Such findings are highly applicable and display two main implications. First of all, since numerous countries have temporarily suspended the enforcement of air pollution regulations in response to the economic shutdowns imposed by the pandemic, severe concerns may arise regarding the indirect impact of stimulus policy stance on the virus spread in highly polluted areas [106]. Conversely, our results highlight the necessity to pursue air quality enforcements to avoid counter-expected consequences including the loss of the public health benefits drawn from the recursive lockdowns and additional heart- and lung-related diseases. Therefore, adequate environmental health measures aiming at lowering the concentration of atmospheric pollutants in targeted localities could slow down the diffusion of COVID-19 across the most vulnerable population. In second, going one step further, the present inferences not only corroborate the polluting particles-health status literature, but also underscore that the COVID-19 crisis revealed an additional fossil fuel-related cost, underestimated so far in urban areas. Linking this new health externality (i.e. COVID-19 spread and lethality) to fossil fuels externalities should help to estimate its effective cost borne by society, prior to internalising it within future energy planning and environmental schemes. Thus, there is a crucial need to reconsider the nature of the energy used by power production, urban planning and transport systems, in China and elsewhere. They indeed generate major externalities on the overall society with important economic and social costs on the short- and long-runs, including the above-mentioned one, newly highlighted in this study. In this paper, we are concerned with their influences on health, as this effective cost remains largely excluded from current cost-benefit analyses. Indeed, many sophisticated models do exist to determine air quality, but they tend to be impractical when it turns to assess the health impacts through a wide range of different channels [1]. For instance, the National Resource Council [107] pointed out that cost-benefit assessments conducted by State Energy Offices do not include all market and non-market impacts when dealing with a measurement of the health impact of air pollution. Here, we claim that estimating the entire costs (i.e. including those related to the COVID-19 outbreak) of generating energy-based fossil fuels for transport and electricity purposes is urgent as it could update the current state of knowledge on the social cost of carbon. In doing so, unpriced consequences could be monetised and better internalised into market mechanisms, but also cost-benefit analyses conducted by public entities. This step is crucial to design adequate climate, energy and air quality action plans. More efficient economic and health outcomes are likely to be reached and prevent major distortions in the future.

The economic slowdown resulting from COVID-19 does not replace climate action because the failure to mitigate climate change and reduce air pollution could lead to a more significant number of losses of human lives and economic costs in the next decades. This is a lesson to keep in mind in the fight against atmospheric pollution and the climate crisis.

Future research might involve the application of a more advanced Deep Learning (DL) process on this topic. It is important to multiply the number of case studies worldwide, as it may update the state of COVID-19 knowledge and enable researchers to identify general trends across cities, regions and countries. If data availability allows that, we suggest future authors to conduct ANNs experiments using a larger set of variables, including meteorological factors (temperature, wind speed, humidity, etc.), health status (obesity, diabetes, cancer incidence) and socio-economic groups (low, middle, high incomes). This may not only confirm the present evidence, but also extend them with finer estimations of the nexus between atmospheric pollution and COVID-19-related-deaths.

## Data

The data that support the findings of this study are available from the Corresponding Author, upon reasonable request.

## Figures and Tables

**Fig. A.**
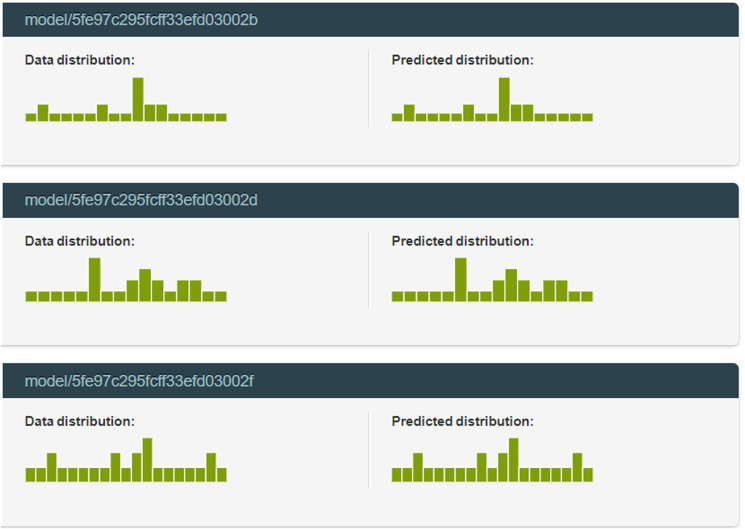
Graphs of data distribution and predicted distribution. *Source*: our elaborations in Oryx 2.0.8 and BML.

**Fig. B.**
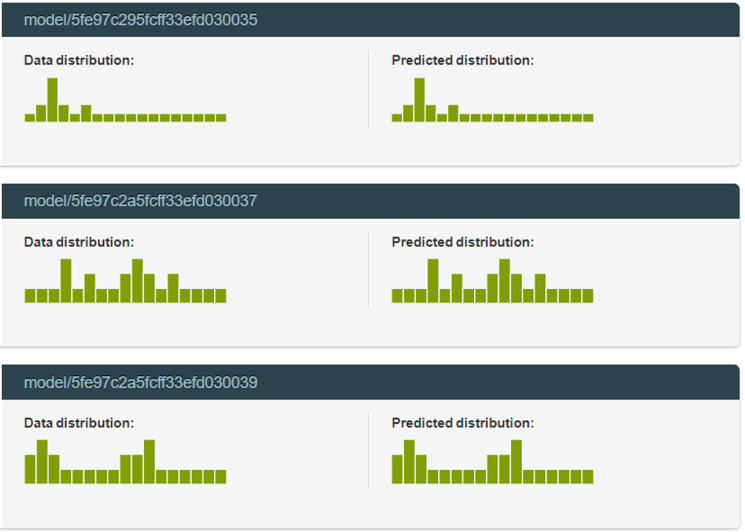
Values of data distribution and predicted distribution. *Notes*: In our model the data distribution is the same as predicted in ML. *Source*: our elaborations in Oryx 2.0.8 and BML.

## Footnotes

1 As it turns to become a major regulatory concern, several emission control policies have been implemented and accompanied by various pollution abatement targets for provinces and cities [25, 43]. For instance, all existing coal power plants have shifted to a new air quality standard in 2014 (GB13223-2011), while industrial and vehicle exhaust emissions have been required to display efficiency gains [44].

2 PM_2.5_: Particulate Matters with diameters smaller than 2.5 micrometres; PM_10_: Particulate Matters with diameters smaller than 10 micrometres; CO_2_: Carbon Dioxide particles.
